# Salvage Therapy with Second-Generation Inhibitors for FLT3 Mutated Acute Myeloid Leukemia: A Real-World Study by the CETLAM and PETHEMA Groups

**DOI:** 10.3390/cancers16234028

**Published:** 2024-11-30

**Authors:** Susana Vives, David Quintela, Mireia Morgades, Isabel Cano-Ferri, Alfons Serrano, Evelyn Acuña-Cruz, Marta Cervera, Marina Díaz-Beyá, Belén Vidriales, José Ángel Raposo-Puglia, Montserrat Arnan, Ana Garrido, Amaia Balerdi, Ana Isabel Cabello, Pilar Herrera-Puente, Josefina Serrano, Rosa Coll, Mar Tormo, Javier López-Marín, Sara García-Ávila, María Soledad Casado, Irene Padilla, Gabriela Rodríguez-Macías, María Calbacho, Ana Puchol, Agustín Hernández, Melissa Torres, Lissette Costilla, Maria Mercedes Colorado, David Martínez-Cuadrón, Jordi Esteve, Pau Montesinos

**Affiliations:** 1 Institut de Recerca Josep Carreras, ICO-Hospital Universitari Germans Trias i Pujol, 08916 Badalona, Spain; dquintelav@iconcologia.net (D.Q.); mmorgades@iconcologia.net (M.M.); 2 Departament de Medicicina, Universitat Autònoma de Barcelona, 08193 Barcelona, Spain; 3 Hospital Universitari i Politècnic La Fe, 46026 Valencia, Spain; isabel_cano@iislafe.es (I.C.-F.); serrano_alfons@gva.es (A.S.); evelyn_acuna@iislafe.es (E.A.-C.); david_martinez@iislafe.es (D.M.-C.); montesinos_pau@gva.es (P.M.); 4 ICO-Hospital Joan XXIII, 43005 Tarragona, Spain; mcervera@iconcologia.net; 5 Hospital Clínic de Barcelona, 08036 Barcelona, Spain; diazbeya@clinic.cat (M.D.-B.); jesteve@clinic.cat (J.E.); 6 IBSAL, Hospital Universitario de Salamanca, 37007 Salamanca, Spain; mbvidriales@saludcastillayleon.es; 7 Hospital Universitario Puerta del Mar, 11009 Cádiz, Spain; josea.raposo.sspa@juntadeandalucia.es; 8 ICO-Hospital Duran i Reynals, 08908 L’Hospitalet del Llobregat, Spain; marnan@iconcologia.net; 9 Hospital de la Santa Creu i Sant Pau, 08025 Barcelona, Spain; agarridod@santpau.cat; 10 Hospital Universitario Cruces, 48903 Barakaldo, Spain; amaia.balerdimalcorra@osakidetza.eus; 11 Hospital Universitario Nuestra Señora de Candelaria, 38010 Tenerife, Spain; acabrodc@gobiernocanarias.org; 12 Hospital Ramón y Cajal, 28034 Madrid, Spain; pilar.herrera@salud.madrid.org; 13 Hospital Universitario Reina Sofía, 14004 Córdoba, Spain; josefina.serrano@iname.com; 14 ICO-Hospital Josep Trueta, 17007 Girona, Spain; rcoll@iconcologia.net; 15 INCLIVA, Hospital Clínico Universitario de Valencia, 46010 Valencia, Spain; tormo_mar@gva.es; 16 Hospital General Universitario de Alicante, 03010 Alicante, Spain; lopez_javmar@gva.es; 17 Hospital del Mar, 08003 Barcelona, Spain; sgarciaavila@psmar.cat; 18 Hospital Universitario de Badajoz, 06080 Badajoz, Spain; marisol.casado@salud-juntaex.es; 19 Hospital Universitario de León, 24008 León, Spain; ipadilla@saludcastillayleon.es; 20 Hospital General Universitario Gregorio Marañón, 28007 Madrid, Spain; gabriela.rodriguez@salud.madrid.org; 21 Hospital Universitario 12 de Octubre, 28041 Madrid, Spain; maria.calbacho@salud.madrid.org; 22 Hospital Universitario de La Princesa, 28006 Madrid, Spain; 23 Hospital Quirón Salud Málaga, 29004 Málaga, Spain; agustin.hernandez@quironsalud.es; 24 Hospital Universitario de Gran Canaria Doctor Negrín, 35010 Las Palmas de Gran Canaria, Spain; m.torresochando@gmail.com; 25 Hospital General San Jorge, 22004 Huesca, Spain; lissettepcb@gmail.com; 26 Hospital Universitario Marqués de Valdecilla, 39008 Santander, Spain; mercedes.colorado@scsalud.es; 27 Departament de Medicina, Universitat de València, 46010 Valencia, Spain

**Keywords:** FLT3 mutations, acute myeloid leukemia, gilteritinib, quizartinib, real world data

## Abstract

Background/Objectives: Patients with relapsed/refractory (R/R) AML with *FLT3* mutation (*FLT3*^mut^) have a dismal prognosis. *FLT3*^mut^ offers a target for therapy in these patients. Gilteritinib (gilter) and quizartinib (quizar) have demonstrated efficacy as single agents in two phase 3 clinical trials. Methods: We retrospectively analyzed the characteristics, treatments, and outcomes of 50 patients with R/R *FLT3*^mut^ AML who received gilter or quizar as monotherapy in 27 Spanish centers before their commercial availability. Forty-four patients were treated with gilter and six with quizar. Results: The median age was 62.5 years, and 52% were women. Most patients presented with *FLT3*-ITD mutations (80%); 46% had refractory disease and 54% had relapsed disease at treatment initiation. First-line treatment was chemotherapy in 80% of patients, with 40% of these also receiving midostaurin. Twenty-five patients (50%) had previously received FLT3 inhibitor, and twenty-eight (56%) had received more than one line treatment before starting gilter/quizar. The rates of complete remission (CR), CR without hematological recovery (CRi), and partial remission were 22%, 18%, and 16%, respectively. The median overall survival (OS) and disease-free survival were 4.74 months and 2.99 months, respectively. We observed a significant improvement in OS in patients who had received only one prior line of therapy compared to those who had received two or more therapies (10.77 months vs. 4.24 months, *p* = 0.016). Multivariate analysis identified failure to achieve CR/CRi, receiving more than one prior line of therapy, age, and white blood cells count as independent prognostic factors for OS. The most common toxicities were febrile neutropenia, liver function abnormalities, and QT interval prolongation. Conclusions: Gilter/quizar monotherapy are effective and tolerable options for patients with R/R *FLT3*^mut^ AML in a real-world setting. Response and toxicity rates are similar to those reported in the phase 3 trials, despite the more heterogeneous nature of the study population.

## Simple Summary

Relapsed/refractory FLT3-mutated acute myeloid leukemia (AML) is associated with a poor prognosis. Mutations in the *FLT3* gene provide a target for therapeutic intervention. Two FLT3 inhibitors, gilteritinib and quizartinib, when used as single agents, have demonstrated efficacy in this context. The aim of our retrospective study was to compare the outcomes of 50 patients diagnosed with *FLT3*-mutated AML treated with gilteritinib or quizartinib monotherapy, with results from phase 3 clinical trials and with other real-world studies. Despite differences among the cohorts, our findings confirm that gilteritinib and quizartinib monotherapy represent effective and tolerable treatment options for patients with relapsed/refractory *FLT3*-mutated AML in real-world settings, with response and toxicity rates consistent with those reported in prior studies.

## 1. Introduction

Relapsed or refractory (R/R) acute myeloid leukemia (AML) has a poor prognosis. In this setting, there are not many available therapies and many of them do not offer significant clinical benefit. Mutations in the *FLT3* gene occur in approximately 30% of patients newly diagnosed with AML [1] and are also observed at relapse [2]. AML with FLT3 gene mutations (*FLT3*^mut^) was initially considered high risk due to the very high relapse rate [3,4]. The European Leukemia Net (ELN) 2022 risk classification [5] changed their risk assignment due to the improvement in overall survival (OS) of *FLT3*^mut^ AML, thanks to the incorporation of *FLT3* inhibitors (FLT3i) [6]. FLT3i offers a targeted therapy at diagnosis and at relapse, in combination [7,8,9,10,11,12] or as single agents [13,14,15]. There are two types of FLT3i: type I are active in internal tandem duplication (ITD) mutations or point mutations in the tyrosine kinase domain (TKD), while type II are active exclusively in *FLT3*-ITD mutations. We distinguish two generations of FLT3i; first-generation inhibitors are less specific due to their inhibition of multiple receptors of tyrosine kinase, while second-generation inhibitors are more selective and potent against *FLT3*, but do not target downstream *FLT3* or signaling pathways [16]. The latter have demonstrated activity in inducing clinical remissions as monotherapy [17,18,19]. Only two second-generation FLT3i, gilteritinib and quizartinib, have demonstrated their effectiveness in monotherapy in two phase 3 clinical trials (ADMIRAL and QuANTUM-R, respectively) when they were used in a R/R *FLT3*^mut^ AML setting [13,14]. In these trials, quizartinib (second generation, type II inhibitor) and gilteritinib (second generation, type I inhibitor) showed an improvement in the median OS compared with the standard of care (SC): 6.2 months vs. 4.7 months (*p* = 0.02) and 9.3 months vs. 5.6 months (*p* = 0.0013), respectively. Considering these results, in different countries, early access programs for these two drugs were started before the regulatory agencies approval [20,21,22,23]. However, few data have been published yet in the use of these FLT3i in the real-world setting.

Herein, we analyze the characteristics, treatments, and outcomes of 50 patients with R/R *FLT3*^mut^ AML who received gilteritinib or quizartinib (gilter/quizar) before its commercialization (i.e., early access programs) in Spain, which were reported to the PETHEMA and CETLAM registries. 

## 2. Methods

This retrospective study included all patients receiving monotherapy with gilter/quizar for R/R *FLT3*^mut^ AML between December 2016 and November 2022, in 27 Spanish centers. Patients were diagnosed with AML according to the WHO 2016 criteria [24] and the prognostic risk was determined by the ELN 2017 classification [19]. All patients were older than 18 years old, diagnosed with R/R *FLT3*^mut^ (ITD or TKD) AML and signed informed consent that was obtained in accordance with the Declaration of Helsinki, allowing for the collection of clinical data in the anonymized database of PETHEMA and CETLAM Cooperative Groups. Bone marrow samples were screened for *FLT3* mutations at a central or local laboratory as per clinical routine. The laboratories used polymerase chain reaction-base assay that was modeled on published methods. *FLT3* mutations were considered to be present if the mutant to non-mutant allelic ratio was at least 0.05%. AML patients who received gilteritinib or quizartinib in other situations, for instance, maintenance in first complete remission (CR), were excluded. Primary refractory AML and relapse were defined according to ELN 2017 criteria [25]: refractory was defined as a failure to achieve CR or CR with incomplete hematologic recovery (CRi) after one or two courses of induction with intensive chemotherapy; and relapse was defined as bone marrow blasts ≥ 5% or reappearance of blasts in the blood, or development of extramedullary disease. For patients who received frontline therapy with hypomethylating agents (HMA), refractory disease was defined as failure to achieve CR or CRi following three cycles of therapy, and relapse was defined according to the ELN 2017 criteria.

Inclusion criteria was not limited to first relapse; patients in second salvage or later were also included. Previous treatments with FLT3i were allowed if they were not administered as single agents for R/R AML. The most frequent treatments included intensive chemotherapy (IC) (3 + 7 schedule, FLAG-Ida or high dose cytarabine) with or without FLT3i (midostaurin, sorafenib, crenolanib or quizartinib), HMA and allo-stem cell transplantation (allo-SCT). Gilteritinib was administered once-daily (120 mg) in 28-day cycles, and patients not achieving CR/CRi after one or two cycles could be escalated to 200 mg/day. Quizartinib was administered 60 mg orally once-daily in 28-day cycles, with dose adjustment in those receiving concurrent strong CYP3A inhibitors. Treatment duration was until documentation of a lack of clinical benefit or the appearance of unacceptable toxic effects. 

The outcome measures were overall response rate (ORR), median probability of OS and disease-free survival (DFS). The ORR included CR, CRi, and PR rates. Response to FLT3i was assessed at the end of cycle 1 (first day of cycle 2) and then when deemed appropriate by the physician, based on the response achieved. OS was calculated from the date of start gilter/quizar treatment to the date of last follow-up or death. DFS was measured from the date of response achieved after gilter/quizar treatment to the date of relapse/progression, last follow-up or death. The toxicity was graded in accordance with the Common Terminology Criteria for Adverse Events (CTCAE), version 5.0 [26].

The patients’ characteristics at diagnosis and at the start of treatment with gilteritinib or quizartinib were described as median and range (for quantitative variables) and frequency and percentages (for categorical variables). To assess the difference between some paired continuous variables (diagnosis—R/R), the non-parametric Wilcoxon test was used. OS and DFS were plotted by the Kaplan–Meier method and compared by the log-rank test. A binary logistic regression model was used to identify prognostic factors for CR/CRi rate and ORR. Multivariable analysis for OS was performed by Cox proportional hazards regression model (considering response and transplantation after gilter/quizar as a time-dependent covariates). All analyses were performed using SPSS (v.24) and R (4.2.0) software. Two-sided *p* values of <0.05 were considered statistically significant.

## 3. Results

### 3.1. Patients and Treatment

Fifty patients with R/R *FLT3*^mut^ AML between January 2016 and December 2021 were included. The main characteristics at the front-line are shown in Table 1. 

Most patients (40/50) received frontline IC (anthracycline plus cytarabine) and in 20/40 cases, IC was combined with midostaurin. Ten out of fifty patients were treated upfront with HMA (nine patients with azacitidine and one patient decitabine). The CR + CRi rate to the frontline treatment was 56% (negative minimal measurable disease was observed in 15 out of 20 patients in whom data are available, 14 of them treated with IC). Allo-SCT was performed in first remission to 4 out of 28 patients. 

At relapse or lack of response to initial treatment, gilter was administered in 44 patients and quizar in 6 patients. Twenty-eight patients (56%) received a salvage therapy prior to gilter or quizar monotherapy initiation. Most of them had been enrolled in clinical trials with or without FLT3i combinations (*n* = 5 midostaurin, *n* = 3 crenolanib vs. placebo, *n* = 3 quizartinib and *n* = 1 sorafenib). The CR + CRi rate for first salvage therapy was 48% (12/25 patients, three not evaluable). Allo-SCT was performed in second remission in 8 out of 12 patients.

The median time from initial AML diagnosis to FLT3i initiation as single agents was 11 months [range, 1.43–44.7]. At the time of gilter/quizar salvage, 23 patients (46%) were refractory to previous AML line (5 to first line treatment, 11 to second line, and 7 to more than 2 lines) and 27 patients (54%) were relapsed AML (17 after first line treatment, 7 after 2 lines, and 3 after more than 2 lines). Overall, 50% of patients had received at least one FLT3i (*n* = 14 midostaurin, *n* = 2 crenolanib or placebo, *n* = 2 sorafenib, *n* = 1 quizartinib), and 6 patients received 2 or more prior FLT3i (*n* = 3 midostaurin and quizartinib, *n* = 2 midostaurin and sorafenib, *n* = 1 midostaurin and crenolanib or placebo). 

The main characteristics at the time of starting gilter/quizar are shown in Table 1. Comparing to newly diagnosed AML, patients in R/R had lower WBC and LDH (21.7 × 10^9^/L vs. 5.1 × 10^9^/L and 528 U/L vs. 285 U/L, *p* < 0.001, respectively). In addition, cytogenetic clonal evolution was demonstrated in R/R status in 8 out of 37 patients studied. 

### 3.2. Outcomes

**Figure 1:**
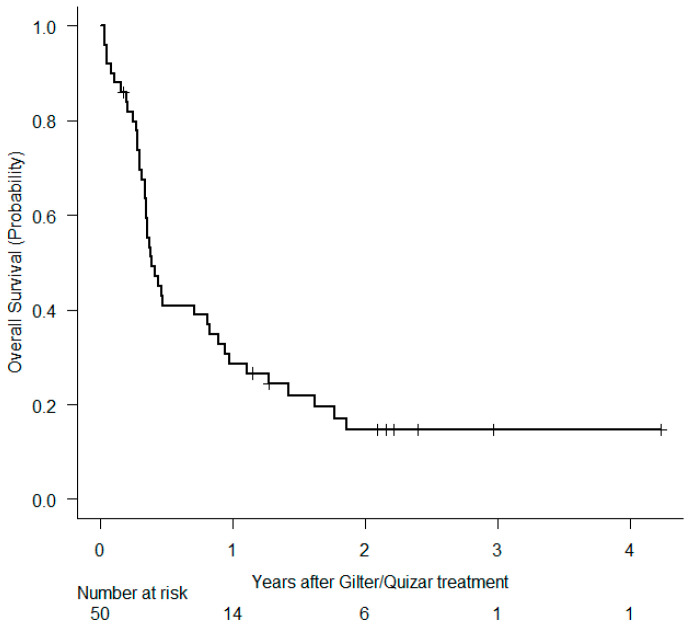
Overall survival for the whole series.

**Figure 2:**
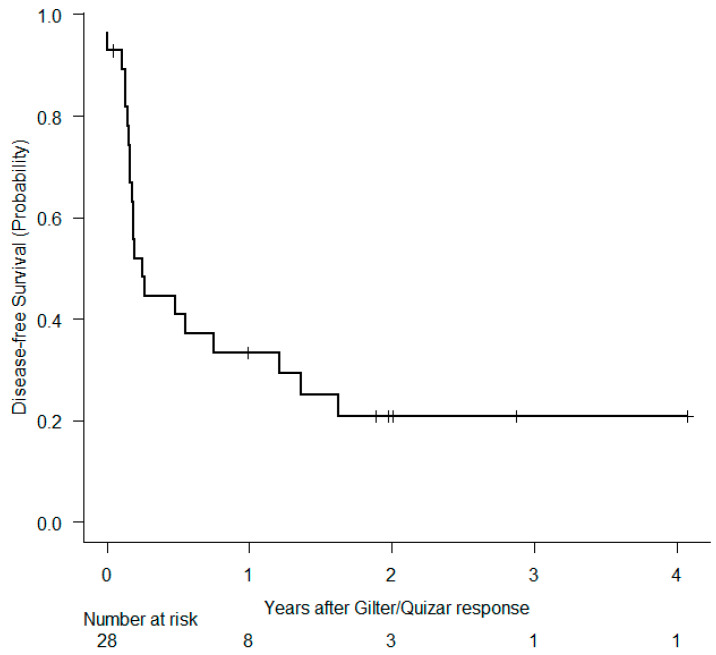
Disease free survival.

**Figure 3:**
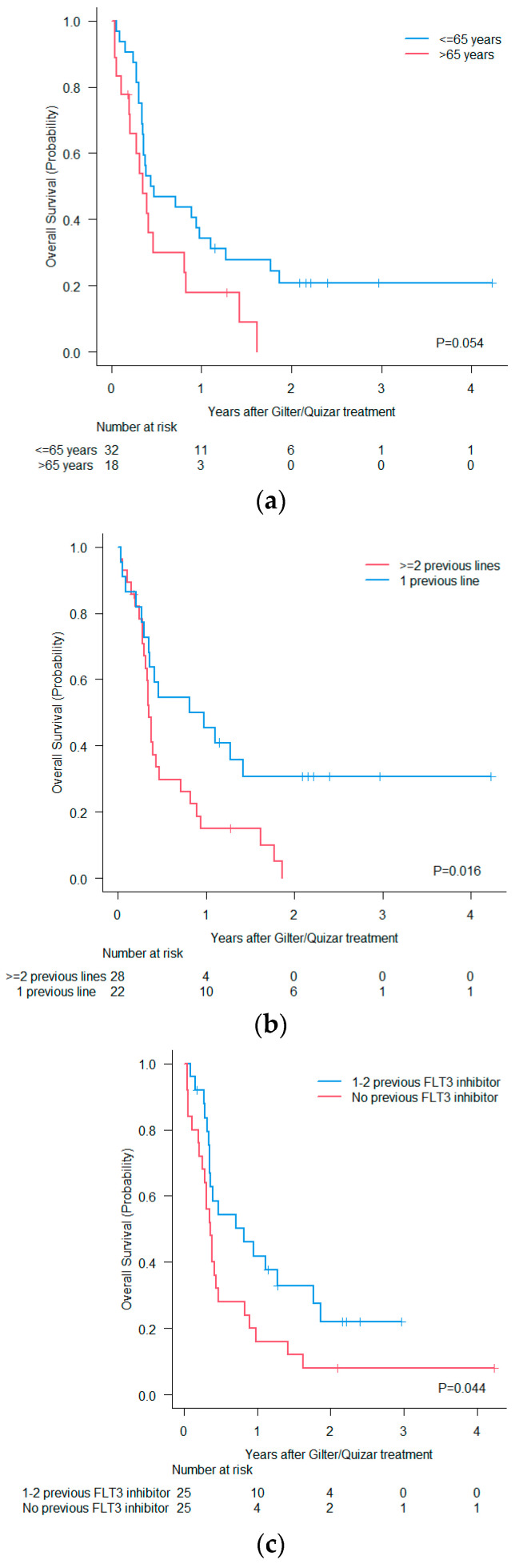

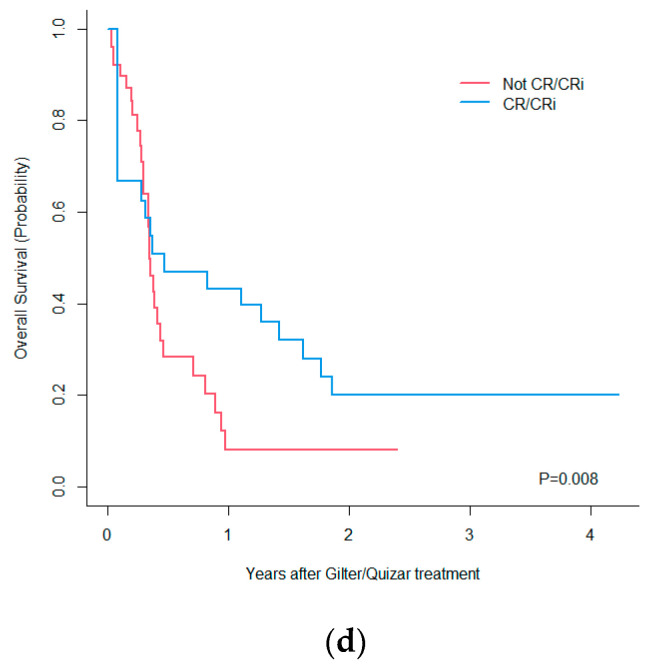
Overall survival by subgroups (**a**): age, (**b**): pre-treatment lines, (**c**): previous exposure to FLT3i, (**d**): response rate as a time-dependent covariate.

Forty-three patients started with gilter 120 mg (standard dose) and one patient 80 mg due to baseline toxicity. Two patients received quizar 30 mg (recommended initial dose), and four patients 20 mg (concomitant treatment with posaconazole or voriconazole). The median number of administered cycles with gilter and quizar was 3 cycles [range, 1–38] and 4.5 cycles [range, 3–55], respectively. After a median of follow up time of 2.16 years [range, 0.18–4.23] there are nine patients alive and under treatment. Out of 41 death events, 24 (58%) were attributable to leukemia, 13 (32%) were caused by infection (3 patients in CR), 2 hemorrhages (5%) and 2 (5%) acute graft-versus host disease. The percentage of patients who reached CR, CRi and PR was 22%, 18%, and 16%, respectively (CR/CRi 40%, ORR 56%). Two patients died before treatment evaluation due to infections. Ten patients (20%) underwent an allo-SCT, 7 of them in CR/CRi, 1 in PR, and 2 with progressive disease. Median OS and DFS in the whole cohort were 4.74 months [95% CI 4.10–9.92] and 2.99 months [95% CI 1.94–14.53], respectively (Figure 1 and Figure 2). Median OS in patients ≤65 years of age trended higher vs. >65 years (median OS 5.46 months vs. 4.21 months, *p* = 0.054) (Figure 3a). We also compared OS between patients who had received only one previous line vs. two or more prior lines, and we demonstrated significantly better outcomes in patients with less prior treatment (10.77 months vs. 4.24 months, *p* = 0.016) (Figure 3b). Comparing OS by number of prior FLT3i, we did not find differences. It should be noted that patients who had not received a prior inhibitor (n = 25) had a significantly worse OS than patients who did receive (4.31 months vs. 9.76 months, *p* = 0.044) (Figure 3c). Patients who achieved CR/CRi (n = 20) had a 6-month OS of 47% (95% CI, 10–78%) vs. 28% (range, 14–45%) in those not achieving CR/CRi (Figure 3d). 

In a univariate analysis for response, relapsed AML was associated with higher probability to achieve CR/CRi and ORR compared to refractory AML (OR 3.051, 95% CI 0.921–10.114, *p* = 0.068 and 3.694, 95% CI 1.139–11.978, *p* = 0.029). A higher allelic ratio (*FLT3*-ITD ≥ 0.5) was a favorable factor for CR/CRi (OR 6.0, 95% CI 1.059–34.003, *p* = 0.043). Age, ECOG PS, WBC count, ELN prognostic risk category, previous treatment with FLT3i, number of previous treatment lines (1 vs. 2 or more), time from diagnosis to gilter/quizar treatment, and previous allo-SCT did not significantly impact the probability of CR/CRi.

In the univariate analysis for survival, age > 65 years old at the time of R/R, higher WBC count, no prior FLT3i exposure, >1 previous therapies, and not achieving CR/CRi with gilter/quizar were associated with worse OS (Table 2). In a multivariable model, not achieving CR/CRi with gilter/quizar, >1 prior therapies, age and WBC count (as continuous variables, increased risk per unit) were independent prognostic factors for OS (Table 2). 

### 3.3. Toxicity

Thirty-five patients (70%) experienced drug-related toxicity during the treatment (4/6 with quizar and 31/44 with gilter). The most frequent were febrile neutropenia (n = 21), liver impairment (n = 10), and QT interval prolongation (n = 7, 5 gilter and 2 quizar). No clinically significant events of torsade de pointes or arrhythmia were reported. All related toxicities are shown in Table 3. There were two deaths attributed to FLT3i toxicity (both infections). Due to the different toxicities, mainly QT interval prolongation and febrile neutropenia, some patients had dose reduction. However, no patient required treatment discontinuation due to drug-related serious adverse events.

## 4. Discussion

In this study, we report a real-world experience including the main characteristics and outcomes of patients with R/R *FLT3*^mut^ AML treated with second generation FLT3i agents (gilter or quizar) as monotherapy. Access to these drugs was available due to early access programs in Spain. Our results are consistent with those observed in two phase 3 clinical trials with oral inhibitors of FLT3 as a single agent, gilteritinib and quizartinib [13,14]. 

Compared to the phase 3 trials (ADMIRAL and QuANTUM-R), our study population includes patients with relapsed or refractory AML after a second or subsequent lines of treatment (28 out of 50 patients received ≥2 prior therapies). We also included 25 patients (50%) with a history of prior FLT3i exposure, and 6 patients had received two prior FLT3 inhibitors; in the ADMIRAL trial only 5.7% of patients had received midostaurin in combination with IC in the frontline setting, while in the QuANTUM-R those patients were excluded. The QuANTUM-R cohort was the youngest (median age 55 years vs. 62 years in ADMIRAL and 62.5 years in our study) and they only included *FLT3*-ITD^mut^. The proportion of adverse risk cytogenetics was higher in our cohort compared to the QuANTUM-R and ADMIRAL studies (43% vs. 9% and 10.5%, respectively). It should be noted that the Spanish Ministry of Health has excluded the high cytogenetic risk patients form reimbursement of gilter (presumably based of sensitivity analyses of the ADMIRAL trial). When we compare our study population with the real-world data published with Gilteritinib from USA [20], France [21], Israel [22], and Turkey [23]: the median age was 58.3, 65.2, 61, and 55 years old (USA, France, Israel, and Turkey, respectively) and 62.5 years old in our study; the percentage of patients receiving prior FLT3i was 100, 50, 40, and 40.1% (USA, France, Israel, and Turkey, respectively) and 50% in our study; and patients who had received more than one previous line of treatment were included in all the studies. Despite the differences in the reported real-world cohorts, the observed outcomes have been comparable to those published by the phase 3 clinical trials. Rates of overall response (CR, CRi and PR), subsequent allo-SCT and median OS in the current study vs. QuANTUM-R and ADMIRAL were 56% vs. 69% and 67.6%; 20% vs. 32% and 25.5%, and, 4.74 months vs. 6.2 months and 9.3 months, respectively. In real life we observed a worse median OS, but when we analyzed the results excluding patients who received more than one line of treatment (*n* = 28), the median OS was 10.77 months (range, 3.62–NA). If we compare our results with real-world data from larger US and French studies (*n* = 113 and *n* = 140) the CR rate and median OS were more similar (22% vs. 22.1% and 16.9%, and 4.74 months vs. 7 and 6.4 months, respectively) (Table 4).

The prognostic factors that may affect the achievement of CR/CRi or OS are controversial. Achieving a CR and undergoing an Allo-SCT were independent favorable prognostic factors to improve OS in two [21,22] real-world series. We confirm that achieving a CR/CRi is a favorable prognostic factor for OS, but also lower age, WBC count and number previous treatment lines were independent factors. When we focus on the analysis of *FLT3* mutations, we observe, as in the ADMIRAL trial, that allelic ratio ITD *FLT3*^mut^ ≥ 0.5 was a favorable factor for CR/CRi, TKD mutation was associated with worse outcomes in our series, but this was not an independent prognostic factor. There are controversial data about gender impact survival in this population. In the ADMIRAL trial, female gender was associated with a significant HR for death of 0.57 (0.40–0.82), contrary to what they found in the French study where the HR was 1.61 (1.07–2.42, *p* = 0.02); and in our study, gender did not affect in CR/CRi rate or OS. 

The first-line use of FLT3i may contribute to cross-resistance to single agents [27]. Perl et al. [28], retrospectively, compared clinical outcomes in patients with R/R *FLT3*^mut^ AML in the CHRYSALIS [29] (multicenter, first-in-human, open label, phase 1/2 trial which assessed the safety, tolerability, and pharmacokinetic effects of gilteritinib in R/R *FLT3*^mut^ AML) and ADMIRAL trials who received prior midostaurin or sorafenib against those without prior FLT3i exposure. Similarly high rates of composite complete remission (CRc) were observed in patients who received FLT3i before gilteritinib (CHRYSALIS, 42%; ADMIRAL, 52%) and those without (CHRYSALIS, 43%; ADMIRAL, 55%). Regarding OS, no significant differences were observed regardless of whether or not they had previously received a FLT3i (CHRYSALIS, OS 7.2 months vs. 7.5 months; ADMIRAL, OS 8.7 months vs. 9.5 months). In the current study, there were 25 patients previously treated with FLT3i, 19 patients received one inhibitor, and 6 patients received two inhibitors. It should be noted that prior exposure to one or more FLT3i did not affect OS in our study; although this variable was significant on univariate analyses, it was not on multivariate analysis. To interpret these results, we have analyzed the clinical and demographic characteristics of these patients and the only difference between the groups (previously treated with FLT3i vs. not treated) was age. The mean age at R/R episode was significantly higher among unexposed patients (62.9 vs. 53.2 years; *p* = 0.02). In multivariate analyses from the French real world-data [30] prior treatment with midostaurin and prior treatment with other FLT3i were not prognostic factors associated with response. Contrarily, Yilmaz et al. [31] published the MD Anderson Cancer Center retrospective experience of the response rates to sequential FLT3i exposure, and they observed that the response rate dropped progressively with sequential exposure to FLT3i. Additionally, in their experience, in all settings, CRc rates were higher with FLT3i based combinations compared with a single agent. 

Our study affirms the efficacy and safety of second generation FLT3i in R/R *FLT3*^mut^ AML. The most frequent side effects were hematological. Non-hematological side effects were sporadic and controlled with temporary suspension and dose adjustment. There were few cases of QTc interval prolongation in all real-world studies (no torsade de pointes) and differentiation syndrome was only described in the French study [21]. 

This study has few limitations, which include a retrospective design and the relatively small number of patients. Bone marrow sample evaluation and diagnosis was performed by different pathologist from all participant centers, and mutational data demonstrating mechanisms of resistance to FLT3i were missing. However, although our study population includes heavily pretreated patients (in comparison to the ADMIRAL and QuANTUM-R), we reproduced similar data from these phase 3 clinical trials.

## 5. Conclusions

In conclusion, in this multicenter real-world study, second generation FLT3i (gilter/quizar) are effective and well-tolerated. Our analysis shows that patients previously treated with FTL3i also respond to treatment. Nevertheless, outcomes with single agents are suboptimal and further improvement is needed, especially in patients treated in second salvage or beyond. Clinical trials with new strategies are urgently needed for patient’s R/R *FLT3* mutated AML.

## Figures and Tables

**Table 1:** Demographic and clinical characteristics of patients at diagnose and relapse/refractory status.

		Newly Diagnosis *n* = 50	R/R Status *n* = 50
Age	Median [range]	61.5 [22–81]	62.5 [25–81]
Age	≤65 years	34 (68%)	32 (64%)
	>65 years	16 (32%)	18 (36%)
Sex	Male	24 (48%) 26 (52%)	
	Female		
ECOG PS	0	22/48 (46%)	17/48 (35%)
	1	21/48 (44%)	20/48 (42%)
	2	4/48 (8%)	10/48 (21%)
	3	1/48 (2%)	1/48 (2%)
WBC, ×10^9^/L	Median [range]	21.7 [0.4–343.7]	5.1 [0.1–140]
ELN 2017 classification	Wild-type *NPM1* and *FLT3*-ITD^high^	13/47 (28%)	10/38 (31%)
	Mutated *NPM1* without *FLT3*-ITD or with *FLT3*-ITD^low^	11/47 (23%)	5/38 (18%)
	Mutated *NPM1* and *FLT3*-ITD^high^	10/47 (21%)	12/38 (25%)
	Wild-type *NPM1* without *FLT3*-ITD or with *FLT3*-ITD^low^	9/47 (19%)	7/38 (13%)
	Mutated *RUNX1*	1/47 (2%)	1/38 (3%)
	t(6;9)(p23;q34.1); DEK-NUP214	1/47 (2%)	1/38 (3%)
	t(8;21)(q22;q22.1); RUNX1-RUNX1T1	1/47 (2%)	1/38 (3%)
	inv(3)(q21.3q26.2) or t(3;3)(q21.3;q26.2);GATA2,MECOM (EVI1)	1/47 (2%)	1/38 (3%)
ELN 2017 risk stratification	Favorable	10 (20%)	4/44 (9%)
	Intermediate	21 (42%)	21/44 (48%)
	Adverse	19 (38%)	19/44 (43%)
*FLT3*-ITD	Negative	14 (28%)	9/45 (20%)
	Positive	36 (72%)	36/45 (80%)
Allelic Ratio *FLT3-*ITD	<0.5	11/36 (31%)	12/34 (35%)
	≥0.5	25/36 (69%)	22/34 (65%)
* NPM1 *	Negative	29 (58%)	23/41 (56%)
	Positive	21 (42%)	18/41 (44%)
*FLT3*-TKD	Negative	37/46 (80%)	31/39 (79%)
	Positive	9/46 (20%)	8/39 (21%)
Bilirubin, mg/dL	Median [range]	0.58 [0.25–3]	0.44 [0.17–6]
Creatinine, mg/dL	Median [range]	0.88 [0.46–2.99]	0.76 [0.42–1.36]
LDH, U/L	Median [range]	528 [155–3795]	285 [98–10,840]
Urates, mg/dL	Median [range]	4.8 [1.2–25]	3.9 [1–10]
Clonal evolution	Yes	NA	8/37 (22%)
	No	NA	29/37 (78%)

R/R: relapse/refractory, ECOG PS: Eastern Cooperative Oncology Group performance-status, WBC: white blood cells, ELN: European Leukemia Net, ITD: internal tandem duplication, TKD: tyrosine-kinase-domain, LDH: lactate dehydrogenase.

**Table 2:** COX regression analysis for overall survival in patients treated with Gilter/Quizar.

Univariate Analisys				
Variable		*n*	OS, HR (95% CI)	*p*
Age R/R	Continuous	50	1.021 (0.996–1.047)	0.096
	≤65 years	32	Reference	0.058
	>65 years	18	1.863 (0.979–3.547)	
Sex	Female	26	Reference	0.581
	Male	24	1.189 (0.644–2.195)	
ECOG PS R/R	0–1	37	Reference	0.447
	≥2	11	1.324 (0.642–2.728)	
WBC count R/R	Continuous	48	1.015 (1.006–1.024)	**0.001**
	<10 × 10^9^/L	32	Reference	**0.001**
	≥10 × 10^9^/L	16	3.241 (1.622–6.479)	
Type of R/R	Relapse	27	Reference	0.331
	Refractory	23	1.358 (0.733–2.514)	
ELN 17 risk stratification	Favorable/Intermediate	25	Reference	0.157
	Adverse	19	1.612 (0.832–3.121)	
*FLT3-*ITD^mut^	Negative	9	Reference	0.797
	Positive	36	1.115 (0.489–2.541)	
Allelic ratio *FLT3*-ITD	Continuous	32	1.149 (0.872–1.515)	0.323
	<0.5	12	Reference	0.873
	≥0.5	22	0.939 (0.434–2.031)	
*FLT3*-TKD^mut^	Negative	31	Reference	0.426
	Positive	8	1.412 (0.604–3.300)	
* NPM1 *	Positive	18	Reference	0.367
	Negative	23	1.391 (0.679–2.848)	
Previous FLT3i exposure	Yes	25	Reference	**0.048**
	No	25	1.872 (1.005–3.487)	
Number of prior therapies	1	22	Reference	**0.019**
	≥2	28	2.185 (1.138–4.196)	
Previous Allo-SCT	No	38	Reference	0.612
	Yes	12	1.203 (0.589–2.456)	
Subsequent Allo-SCT	No	40	2.028 (0.758–5.422)	0.159
	Yes	10	Reference	
CR/CRi after Gilter/Quizar	No	30	3.727 (1.497–9.276)	**0.008**
	Yes	20	Reference	
ORR post Gilter/Quizar	No	22	1.568 (0.774–3.176)	0.212
	Yes	28	Reference	
Time from newly diagnosed AML to Gilter/Quizar	Continuous	50	1.015 (0.987–1.043)	0.310
	≥12 months	22	Reference	0.971
	<12 months	28	1.012 (0.544–1.880)	
**Multivariable Analisys**				
** Factor **		***n***	**OS,** **HR (95% CI)**	***p***
Age at R/R (increase risk per year)		48	1.035 (1.008–1.063)	**0.011**
WBC count at R/R (increase risk per 1 × 10^9^/L)		48	1.013 (1.004–1.022)	**0.006**
Number of prior therapies (>1 line)		48	2.225 (1.124–4.404)	**0.022**
No CR/CRi after Gilter/Quizar		48	5.170 (1.999–13.638)	**0.001**

OS: overall survival, R/R: relapse/refractory, ECOG PS: Eastern Cooperative Oncology Group performance-status, WBC: white blood cells, ELN: European Leukemia Net, ITD: internal tandem duplication, TKD: tyrosine-kinase-domain, FLT3i: FLT3 inhibitors, Allo-SCT: allogeneic stem cell transplant, CR: complete remission, CRi: complete remission with incomplete hematologic recovery, ORR: overall response rate.

**Table 3:** Gilter/Quizar related adverse events during treatment.

	Any Grade	Grade ≥ 3	FLT3i Discontinuation	Dose Reduction
Febrile neutropenia	21/49 (43%)	15/15	8/21	4/21
Liver toxicity	10/49 (20%)	3/6	2/10	1/10
QTc interval prolongation	7/49 (14%)	2/7	2/7	4/7
Myelotocicity	3/49 (6%)	3	1/3	1/3
Rash	2/49 (0.4%)	0	0	0
Sweet and Differentiation syndrome	1/49 (0.2%)	0	0	0
Pulmonary edema	1/49 (0.2%)	1/1	0	0
Syncope	1/49 (0.2%)	1/1	1/1	0
Diarrhea	1/49 (0.2%)	1/1	0	0
VVZ infection	1/49 (0.2%)	0	0	0

FLT3i: FLT3 inhibitor, VVZ: virus varicella zoster.

**Table 4:** Comparative real-world data studies with gilteritinib.

Real-World Data	*n*	Age, Years (Range)	Prior FLT3i Exposure (%)	Number of Prior Therapies	Duration of Gilteritinib	CR (%)	Median OS (Months)	SAE	Mortality at 30 and 60 Days (%)	Prognostic Factors OS
Numan Y et al. *Am. J Hematol*. **2022** [20]	113 *	58.3 (18–92)	100	NA	4.6 months (0–25)	22.1	7 (SD ± 7)	NA	NA	Achieve CR Underwent SCT
Dumas PY et al. *Leukemia*. **2023** [21] **	140	65.2 (23.1–86.1)	50	29.3% (≥2)	NA	16.9	6.4 (IQR, 3.2–14.7)	Thrombocytopenia (51.4%), neutropenia (48.9%), anemia (40.3%).	5.0 and 12.9	Female gender Adverse cytogenetic risk Underwent SCT
Shimony S et al. *Ann Hematol*. **2022** [22]	25	61 (IQR1–3, 47–73)	40	2 (1–3)	2 cycles (1–34)	48	8 (0–16.2)	Thrombocytopenia (20%), hepatic (24%)	8.0 and 28.0	Achieve CR Prior IC
Dogu MH et al. *Mediterr J Hematol Infect Dis*. **2023** [23]	17	55 (27–73)	41.1	1 (1–5)	8.5 months (1–21)	64.7	355.5 days (21–905)	Hypocalemia (41.2%), anemia (41.2%)	5.9 and 11.8	Febrile neutropenia Peripheral edema

FLT3i: FLT3 inhibitor, CR: complete remission, OS: overall survival, SAE: Serious adverse events, SD: standard deviation, NA: not available, SCT: stem cell transplantation, IQR: interquartile range, IC: intensive chemotherapy. * 71 patients received gilteritinib alone and 42 patients in combination therapy. ** The data provided is from Cohort B in the French study.

## Data Availability

Data available upon request due to restrictions (study approved by an ethics committee and subject to the study’s own investigations under informed consent signed by the included patients). The data presented in this study are available upon request from the corresponding author for ethical reasons.
